# Inflammatory Bowel Disease Therapeutics: A Focus on Probiotic Engineering

**DOI:** 10.1155/2022/9621668

**Published:** 2022-01-17

**Authors:** Jayshree Mishra, Madyson Stubbs, Longxiang Kuang, Nitza Vara, Priyam Kumar, Narendra Kumar

**Affiliations:** ^1^Department of Pharmaceutical Sciences, Irma Lerma Rangel College of Pharmacy Texas A&M Health Science Center, Kingsville, TX 78363, USA; ^2^Santa Gertrudis Academy High School, Kingsville, Texas, USA

## Abstract

Inflammatory bowel disease (IBD) is a chronic inflammatory condition of gastrointestinal (GI) tract with dysregulated mucosal immune functions and disturbed commensal ecosystem of the intestinal lumen. IBD is categorized into two major subsets: Crohn's disease (CD) and ulcerative colitis (UC). Though advent of biologics has shifted the treatment with relatively longer remission compared to small molecule pharmaceuticals, patients still suffer from long-term complications. Since gut-microbiome is now accepted as another human organ holding potential for long-lasting human health, probiotics, and its engineering hold great promises to treat several previously untreatable chronic inflammatory conditions including IBD. Several emerging biological engineering tools have unlimited potential to manipulate probiotic bacterial system. These can produce useful therapeutic biologics with a goal to either ameliorate and/or treat previously untreatable chronic inflammatory conditions. As gut-microbiome is diverse and vary in different ethnic, geographic, and cultural human population, it will be important to develop vision for personalized probiotic treatment and develop the technology thereof to make personalized probiotic options a reality. The aim of this review paper is to present an overview of the current knowledge on both pharmacological and nonpharmacological IBD treatment modalities with a special emphasis on probiotic strains that are developed through the probiotic engineering. These engineered probiotics contain the most anti-inflammatory cytokines found within the human immune response and are currently being used to treat the intestinal inflammation in IBD for the IBD treatment.

## 1. Introduction to IBD

Inflammatory bowel disease (IBD) is a chronic inflammatory condition of gastrointestinal (GI) tract which results from uncontrolled immune responses to the food antigens and microflora present in the intestinal lumen [1]. IBD is subcategorized into two major subsets, ulcerative colitis (UC) and Crohn's disease (CD), and the onset of the disease is normally diagnosed in the 20s-30s but can be seen in children and adolescents as well [2]. Seventy percent of patients with active flare-ups may have recurrent episodes of flare-ups at some point during the following year that leads to a 30% chance of relapse with patients in remission during the entirety of the preceding year [3].

IBD in children and adolescents is relatively uncommon compared to other gastrointestinal disorders and childhood diseases. Out of 100,000 children younger than 15, less than 2-3 cases are usually seen. According to American Colleges of Gastroenterology, most true cases of pediatric IBD are seen in the 15-19 years age range at an average of 16 out of every 100,000 cases [2]. Several factors contribute to the risk of developing or being diagnosed with IBD. Some of the main risk factors include age, socioeconomic status, and stress levels. Individuals over the age of 45 are also at increased risk for IBD. Living in what is considered “Western Society” is also a risk factor mainly attributed to diet and access to healthcare. People of Hispanic heritage and non-Hispanic white origin are also considered risk groups [4]. Stress-inducing life situations and living in suburban areas are also risk factors [5]. Obesity is another risk factor for the development of IBD because obesity affects body's ability to self-regulate toxins and/or inflammatory cytokines, thereby making them prone to chronic low-grade inflammation and IBD [6].

Although the definitive causes for IBD are unknown, several factors are being suspected. Among others, the major factors include genetic predisposition and environmental factors that chronically trigger immune system which attack the self-tissues of the gastrointestinal tract. Though pathophysiology of these interactions are complex and multifactorial, the suspected genetic basis of the disease has been emerging in recent literature [7]. There are at least 163 gene loci that have been identified as having involvement in the IBD, and these loci vary between CD and UC [8]. Now, emerging as a global disease, recent study has stratified IBD evolution into four epidemiological stages, viz., emergence, acceleration in incidence, compounding prevalence, and prevalence equilibrium [9].

In this review paper, we discussed about the current intervention (both pharmacological and nonpharmacological) to IBD with special emphasis on treatment with the engineered probiotics. We elaborated on the IBD therapeutics such as IL-10, IL-35, IL-27, trefoil factors, and TNF-*α* that are engineered through probiotic engineering and found to treat intestinal inflammation with a goal to avoid common complications and side effects from current therapies.

## 2. Current Interventions to IBD

Since there is no cure for IBD, treatment options for CD and UC are aimed at reducing symptoms and improving quality of life. A patient is considered to have refractory IBD if persistent attempts to treat active disease flare-ups fail to reduce symptoms, or when the acute treatment is discontinued, the symptoms return immediately [10].

### 2.1. Pharmacological Interventions

#### 2.1.1. Anti-Inflammatory Drugs

Anti-inflammatory drugs for IBD include corticosteroids and amino salicylates to decrease inflammation and avoid damage to the intestinal tract. Considered as first line treatment for both UC and CD, these oral anti-inflammatories are members of the 5-ASA class of drugs and include mesalamine (Asacol HD, Delizicol), balsalazide (Colazal) and olsalazine (Dipentum). Suppository dosage forms and enemas also exist for some of these medications [11]. Corticosteroids are normally used for more severe cases where side effects can limit length of therapy, especially when chronic treatment is warranted [12]. Budesonide is one such commonly used corticosteroid approved for treatment of UC, though only approved for acute treatment of flare-ups and not for chronic maintenance therapy. Both these treatment options are also the options during pregnancy [12].

#### 2.1.2. Immunomodulators and Biologics

As overactive immune system causes the inflammation and tissue damage in IBD, immunomodulators are being used to reduce the tissue damage. Immunomodulators work in various ways to reduce the release of chemicals such as cytokines, that cause inflammation of intestinal lining. The drugs can be used as monotherapy or in combination with other pharmaceutical agents. Some of these drugs include azathioprine, mercaptopurine, cyclosporine, and methotrexate.

Biologics are also commonly used to reduce inflammatory cytokines and include antitumor necrosis factor-alpha (TNF-*α*) antibodies which act through neutralizing TNF-*α*, an inflammatory cytokine responsible for much of the inflammatory damage seen in IBD. Drugs such as infliximab, adalimumab, and golimumab fall into this category of drugs commonly used to treat IBD. Other drug classes include anti-integrin molecules such as natalizumab and vedolizumab. Though biologics are effective, these drugs can become quite expensive given that they are relatively new to the market, and many of them are still under their original patented brand name formulation [13]. TNF-*β* (+252A/G) polymorphisms showed a significant increase in the frequency of the GG genotype in IBD patients, suggesting a positive association of GG genotype with IBD risk. The genotype GG of TNF-*β* was associated with susceptibility risk to UC but not CD where the frequencies of alleles and genotypes of both TNF-*α* and-*β* polymorphisms are not affected by sex or type of IBD (familial or sporadic) [14].

#### 2.1.3. Antibiotics

Antibiotics are a common addition to treatment regimens for patients with IBD particularly having Crohn's disease. Not only does IBD inherently cause chronic damage of body's first line of defense against infection by causing trauma to gastrointestinal epithelium, but many of the drugs used as therapies in the treatment of IBD such as corticosteroids, anti-inflammatories, and biologics also have side effects that increase a patient's likelihood of contracting secondary infections [15, 16]. Within IBD, there are also certain disease processes that require antibiotics more than others, such as in cases of perianal Crohn's disease. Commonly used antibiotics for IBD include metronidazole and ciprofloxacin [17].

#### 2.1.4. Other Medications and Supplements

There are other medications frequently used to relieve symptoms of IBD. For example, diarrhea or loose stools is one of the most frequent symptoms experienced by IBD sufferers, and there is a common practice to prescribe over the counter (OTC) antidiarrheal medications. Fiber supplements such as psyllium or methylcellulose are often chosen to help alleviate mild to moderate loose stools [18]. Loperamide may be recommended for more severe cases of diarrhea. OTC pain relief may also be recommended for mild pain associated with IBD. Due to the increasing symptoms and possibly exacerbating severity by NSAID medications such as ibuprofen or naproxen, acetaminophen is the most frequently recommended medication [19]. Iron supplements are recommended for patients with chronic bleeding issues and calcium/vitamin D supplements to help reduce risk of secondary IBD-associated osteoporosis [5].

#### 2.1.5. Vaccinations

For patients with moderate to severe IBD, long-term immunomodulators are regularly prescribed where sustained suppression of immune response can leave patients susceptible to bacterial and viral infection. For that reason, age-appropriate vaccinations are recommended including MMR, TDaP, HAV, HBV. HPV, pneumonia, influenza, and herpes zoster [20]. In many instances, patient may have had some of these vaccinations prior to diagnosis with IBD; therefore, having an accurate vaccination history is important for determining proper immunization coverage. It is also recommended to have these vaccinations done prior to initiating immunosuppressive therapy to ensure proper immune response and reduce the risk of secondary infection [21].

### 2.2. Nonpharmacological Interventions

Although pharmacological interventions are preferred, other treatment strategies can be employed for patients with IBD. These strategies include nutritional approaches, apheresis, physical activity/exercise, and psychological strategies. Research on nutritional strategies such as probiotics or certain diets alone is limited and has not been shown to have enough effects on its own to be used as sole treatment. However, these were found helpful in supplementation to medical treatment [22]. Nutritional strategies exercise and psychotherapy are also not used as sole therapies, but in conjunction with other strategies, mainly to improve a patient's quality of life [23].

Another nonpharmacological treatment involves the removal of white blood cells from a patient's blood circulation. There are two different approaches used so far: leukocytapheresis (LCAP) and granulocyte-monocyte-apheresis (GMA). LCAP involves extracorporeal adsorption of lymphocytes, granulocytes, and monocytes while GMA is more selective, only adsorbing activated granulocytes and monocytes/macrophages [24]. While both treatments have excellent safety profiles and have shown to induce and maintain remission in adults with UC, the number of controlled studies is too limited to draw definitive conclusions [22, 24].

#### 2.2.1. Nutrition

In cases of hospitalization where the bowel is severely damaged, it may be necessary to administer special diet either through a feeding tube or parenterally to allow patient receive proper nutrition while letting the bowel rest. This can allow the body to direct more energy towards healing the damaged areas of the bowel and aid in the correction of any problems with malabsorption of nutrients in inflamed areas of tissue. For example, low residue diets generally help reduce the risk of food blocking the colon in patients with stenosis [25].

#### 2.2.2. Lifestyle Changes

It is always important to stress the value of a healthy diet and lifestyle in patients with IBD. It can also afford patients a more concrete hold on the control of their disease and its symptoms. Increased intake of vegetables and fruits and decreased intake of red meat can decrease the number of symptomatic flare-ups. A healthy lifestyle including regular exercise schedule and smart appropriate meal choices is known to improve overall IBD complications [26].

Surgery: surgical intervention is normally opted when other treatment options fail or are no longer effective [27]. Roughly a third of UC patients with symptoms for over 30 years or more require surgery compared to 70% patients with symptoms of CD [28]. Patients with UC can have long-term remission through surgery. This normally consists of removal of the entire rectum and colon and possibly the creation of a stoma attached to an ostomy bag [9]. Surgical procedures for CD on the other hand can help alleviate severe symptoms by removing damaged area of the bowel. This does not, however, provide long-term remission in patient as it does in UC. Often after surgical intervention in CD, there is a recurrence of symptoms later in patient's life. Recent studies show that gastrointestinal surgeries can also lower gut microbiome diversity [29].

#### 2.2.3. Psychological Intervention

Studies hypothesizing stress reduction as a technique for additional control of IBD symptoms have been used with varied results. Several studies show inconclusive or negative correlations between stress reduction and symptomatic amelioration. One such study known as the INSPIRE found evidence to support that stress reduction techniques did not improve overall health outcomes for IBD patients although there was evidence to suggest that quality of life might be generally improved [30].

### 2.3. Shortcomings of Current Interventions

Through since mid-1900s, an increase in the number of drugs has become available to treat IBD, the optimal therapy for IBD is far from reality. Future concepts in IBD trial design with a focus on the role of comparative research and the challenges and pitfalls in undertaking and interpreting the results from such studies are reported [31]. Among the well-known issues encountered in IBD treatment include nonadherence. Nonadherence arises from many factors such as a patients' fear of side effects or different drug routes, to name a few. For example, topical therapy with suppositories/enemas used with corticosteroids and 5-ASAs has been associated with increased nonadherence over oral therapies [32]. Therefore, IBD being complex disease may require diverse and mutually interacting components. This requires complex approaches with the notion for personalized drugs [33].

## 3. Gut Microbiome and IBD

### 3.1. Gut Microbiome and Energy Harvest from Foods

Microorganisms that reside in and on the human body are referred as human microbiota. Microbiome on the other hand refers to the total genes they encode [34]. Human gastrointestinal tract (GIT) has one of the largest surface areas of any organ in the body [35] and is the home to a numbers of species of microbiota [36] ranging from 250 to 400 m^2^. The microbiome of human gut comprises more than 100 trillion microbial organisms that include bacteria, fungi, viruses, and protozoa [37]. Interestingly, the enteric system microbiome in human comprises over 100 times the genes compared to the genome of its human host [38]. Majority of intestinal bacteria belong to 4 phyla, Firmicutes, Bacteroidetes, Proteobacteria, and Actinobacteria, where the healthy human gut has predominantly Firmicutes and Bacteroidetes [39, 40]. Bacterial population also vary throughout the gastrointestinal tract with colon having both the greatest number and diversity of species compared to stomach and small intestine [41]. Gut microbiome plays a key role in several aspects of host physiology including nutrition, immune development, metabolism, protection against infection, and gut homeostasis [42]. Gut microbiome also signifies overall host health maintenance and disease attributions [43]. Therefore, several studies have been conducted to understand the constituents of microbiota using techniques such as 16S rRNA-sequencing and microbial transcriptome analysis. The Human Microbiome Project (HMP) is aimed at finding the relationship between microbiota phenotypes and disease phenotype [44], generated Unified Human Gastrointestinal Genome (UHGG) collection. UHGG collection consisted of 204,938 nonredundant genomes from around 4,644 of human gut prokaryotes [44]. There are over 2000 bacterial species inhabit inside in the gut which is over 10 folds higher than the total body cell number [45]. The collected genomes encoded over 170 million of protein sequences and have profound impact on the pathogenesis of different disease [43]. Dysbiosis of microbiome has been identified as disturbances in their diversity, growth rate, and biocomposition of microbiota [45]. The dysbiosis in the gut is accompanied with many adverse impacts including dysfunctional mucosal epithelial cells, disruption of gut immune homeostasis, and uncontrolled gut inflammation [46].

### 3.2. Gut Microbiome and IBD

IBD is thought to arise from as result of a multitude of environmental and bacterial interactions with immune-mediated factors in a genetically susceptible host. Studies suggest a role of gut microbiome in the pathogenesis of IBD where animal models provided convincing evidence of an altered microbiome and aberrant immune response to the microbiome leading to the development of chronic intestinal inflammation [47]. Analysis of the composition of fecal microbiota through molecular assessment (deep and global) from 108 participants (62 patients with IBD and 46 healthy volunteers) showed that patients with IBD had double the ratio of Firmicutes to Bacteroidetes compared to healthy subjects [48]. Among these, species such as Ruminococcus, Clostridium, and Dorea showed a 1.5-fold increase while Faecalibacterium and Bifidobacterium species numbers a 1.5-fold decrease in patients with IBD. Methanogen numbers on the other hand showed reduction by 4-fold in IBD compared to the healthy controls [48]. Other research showed significant relation between the gut dysbiosis and IBD that also correlated with visceral hypersensitivity [49]. Study also suggests overgrowth of fungi and different bacterial species such as lactose-fermenting Lactobacillus, Streptococcus, Escherichia, and Klebsiella and reduced growth of *Prevotella* in IBD [50]. It is known that a reduction in Bifidobacterium and Lactobacillus leads to short-decreased short chain fatty acid (SCFA) formation led dysbiosis [51]. An increase in inflammatory response led byproducts of respiratory electron acceptors seen in IBD which results in oxidative environment and the overgrowth of *Enterobacteriaceae*, *Escherichia coli*, and *Ruminococcus gnavus* [52]. These indicated oxidative stress as one of the factors promoting gut dysbiosis [52].

### 3.3. Gut Microbiome in IBD Intervention

Among the new frontiers to treat IBD are the modification of gut microbiome by using prebiotics, antibiotics, fecal microbial transplantation, and others. Hence, more in-depth studies are needed to understand the correlation between microbiome ecosystem and IBD. Transfer of proinflammatory microbiota from diseased mice into healthy mice leads to increased levels of inflammation in an otherwise healthy mice [53], and colonization of mice with intestinal microbiota from IBD patients also exacerbates colitis through altering immune responses [47]. Findings also suggest transfer of naive T-helper lymphocytes from healthy mice into mice lacking T and B cells leads to increased symptoms of colitis [48–50].

Genetic markers associated with IBD in human are related to engagement between the immune system and gut microbiota [51]. Furthermore, recent studies have demonstrated a role for specific microbes in driving or suppressing inflammation, predicting response to therapy, and determining the risk for disease recurrence after surgery [52]. Therefore, fecal stream diversion has been an effective strategy in the management of Crohn disease (CD) with remission occurring in the excluded segment of the bowel. After diversion, restoration of continuity and thus reexposure to the fecal stream are associated with postoperative recurrence of CD, and antibiotic therapy using ciprofloxacin and metronidazole has proved useful in remission in certain phenotypes of IBD in patients with perianal CD and pouchitis and metronidazole for the prevention of postoperative recurrence in patients with CD [54].

By assessing the ribosomal rRNA through a fluorescence microscope, the fecal microbiota can be studied and used to determine the fecal microbiota and its relation to diseases. So far, as IBD diagnosis is concerned, both UC and CD have been diagnosed by inspecting patient's fecal microbiota composition. For example, residential bacteria such as *Eubacterium rectale*, *Bacteroides*, and *Faecalibacterium prausnitzii* are present in healthy individuals, contributing up to 40% of the total fecal microbiota. However, in Crohn's disease and diarrhea, the presence of these bacteria are often nonexistent or greatly reduced [55]. *Eubacterium rectale* is vital for the consumption and digestion of potatoes and high amylose corn starches in humans. It also metabolizes resistant starch degraded by *Ruminococcus bromii*, thereby producing SCFA such as propionate and butyrate. This shows resistant starch degradation through symbiotic relationship *R. bromii* and *E. rectale* where SCFA produced are beneficial to host health as they contribute to energy metabolism, inhibit inflammation, and promote colonocyte proliferation [55]. *Faecalibacterium prausnitzii* is also important for gut health as it is a main butyrate producer that enhance the intestinal barrier function [56]. These microbes are significant in their contribution to probiotic production, symbiotics maintenance, and IBD symptom amelioration.

Dysbiosis of gut microbiome can occur in several ways including through exposure to toxins, pathogens, and a change in diet. For example, foodborne pathogens which led inflammation can directly alter gut oxidative state and allow the alteration in microbiota and deteriorate host's barrier function [57]. Additionally, *Mycobacterium paratuberculosis* has been associated with the pathogenesis of CD. However, it is not known if *M. paratuberculosis* causes CD and can be caused by inflammation due to an altered gut environment [58].

### 3.4. Gut Microbiome and Diet in IBD

It was reported that dietary intake has a significant impact on the gut microbiota, which in turn determines health status and diseases pathophysiology including IBD [59]. The intestine has a delicate balance between the beneficial bacteria that secrets vitamins and harmful microbiota that secrets toxic substances [60]. Fibers and live healthy bacteria (probiotics) assist in stabilizing the beneficial microbiota. Moreover, some beneficial large bowel microbiota ferment proteins and fibers to produce SCFA that keeps the integrity of the tissue and act as an energy source [61]. Dietary ingredients can also change the inflammatory state of the intestine, for example, omega-6 fatty acids (from vegetable oils) are proinflammatory while omega-3 fatty acids (from fish) are anti-inflammatory [11]. Research also indicates that while high-carbohydrate consumption may reduce the diversity of gut microbiome, fruits and vegetables may increase microbiome diversity. The consumption of processed food on the other hand may lead to different diseases including IBD due to low micronutrients [62]. Diets also may trigger IBD through epigenic modulation [63].

## 4. Probiotics and IBD

### 4.1. History of Probiotics

Use of probiotic has been recognized since the beginning of mankind where human utilized fermented milk, yogurt, and cheese, utilizing their nutritional benefits [64]. In the early 20th century, Metchnikoff argued that not all microorganisms are detrimental to human health and certain bacteria such as the Streptococcus thermophilus and Lactobacillus delbrueckii species provide benefit by promoting a healthy microflora in the gastrointestinal tract [65]. Metchnikoff's theory was recognized which led to the multibillion dollar industry [66].

### 4.2. Definition and General Principles of Probiotics

The approval for the health claims of different probiotics was formally initiated through the 2001 Joint Expert Consultation of FAO/WHO which recognized the need for guidelines. Thus, probiotics are defined as “*live microorganisms which when administered in adequate amounts confer a health benefit on the host*.” [67]. The evaluation guidelines of these probiotics included microbial identification through phenotypic and genotypic methods, in vitro and in vivo studies, and double-blind randomized and placebo-controlled human trials. The two major genera of probiotics include Lactobacillus and Bifidobacterium where the former is nonspore forming gram-positive rod-shaped aerotolerant or anaerobic and belong to the lactic acid group of bacteria [68] while the latter is nonspore forming mostly anaerobic. Both the genera offer modulation on the intestinal crypt dynamics and protection against pathogens such as rotavirus [69].

True probiotics are in general of human origin and should be free from any vectors or other factors that can transfer antibiotics resistance. A probiotic should also show antagonism to pathogens and stimulate the immune system and demonstrate beneficial effects on the host [70]. Overall, there are four characteristics of an effective probiotics: (a) probiotics should be able to survive the passage through the digestive system, (b) probiotics should be able to attach to the intestinal epithelia and colonize, (c) probiotics should be able to maintain good viability upon successful passage through the intestine, (d) probiotics should be able to utilize the nutrients and substrates in a normal diet, and (e) probiotics should be free from pathogens and should not contain any microbial or nonmicrobial toxic products [70, 71].

### 4.3. Prebiotics

ISAPP defines prebiotics as “a substrate that is selectively utilized by host microorganisms conferring a health benefit” [72]. These are also called colonic food and mostly consists of soluble dietary fibers that promotes growth of the symbiont intestinal microbiota. Major categories of prebiotics include fructans, galacto-oligosaccharides, starch- and glucose-derived oligosaccharides, and other oligosaccharides [73]. There are also noncarbohydrate prebiotics such as cocoa-derived flavanols [74]. Prebiotics are anticipated to stimulate the growth of certain bacteria by supplying energy sources. For example, Bifidobacterium species can ferment starch and fructans and therefore have shown significantly higher growth in infants with the stimulation with starch and fructans [75, 76]. Prebiotics are also reported to be effective in management of IBD. Both IBS and Crohn's disease are reported to have decreased number of Bifidobacteria [77]. Since *Bifidobacteria* is beneficial in suppressing microbiota imbalance in the gastrointestinal tract caused by IBD pathology, clinical trials have suggested consumption of fructo-oligosaccharides as beneficial in improving IBS/IBD symptoms [78]. Study also suggests fructo-oligosaccharides in increasing *Bifidobacteria* populations in patients with Crohn's disease [79]. However, other clinical trials have also shown otherwise from prebiotics in patients with active IBD conditions [80, 81]. These suggest additional but unknown factors may play role in prebiotic outcome in IBD patients.

### 4.4. Probiotic Mechanisms of Action

The overall mechanisms of action for the probiotics are targeted towards two major aspects: (a) changing the composition and function of gut microbiome and (b) promoting intestinal mucosal physiology and immunobiology to facilitate anti-inflammatory response to facilitate wound healing. Probiotic mechanisms of action thus among others include enhancing of the epithelial barrier, increased adhesion of healthy probiotic microbes to intestinal mucosa, inhibition of pathogen adhesion to mucosal surfaces, competitive exclusion of pathogenic microorganisms, production of antimicroorganism substances, and modulation of the immune functions. For example, certain probiotics produce antimicrobial metabolites that suppress the growth of other microorganisms [82], while other (Lactobacillus) promote intestinal integrity and barrier functions, thereby facilitating immune tolerance with decreased bacterial translocation across the mucosal barrier [83].

#### 4.4.1. Reestablishment of Intestinal Symbiosis through the Application of Probiotics

(A) *Competitive exclusion of pathogens*: probiotics facilitate microbial diversity through competitive exclusion where bacteria from probiotic formulations compete with dysbiotic and pathogenic species for receptor present in the gastrointestinal tract [84]. Though regulatory elements and associated specific pathways underlying these effects are largely unknown, some of the mechanisms proposed include creating acidic environment to reduce competition, competing for nutritional sources, and production of bacteriocin or bacteriocin-like substances for competitive exclusion of pathogens [85].

Several mechanisms have been reported through which probiotics facilitate reestablishment of intestinal symbiosis. For example, it was reported that while some probiotics produce antimicrobial peptides or metabolic that suppress the growth of other microorganisms [86, 87], others compete with intestinal pathogenic microbes for receptors or binding sites on the intestinal mucosal surfaces [88]. The impact of probiotics on the composition, diversity, and functions of the gut microbiome has mainly been studies using tools such as targeted microbial culture followed by microbial estimation and metagenomic sequencing. Moreover, studies demonstrating alteration of microbiota following probiotic intervention are very limited. One clinical study demonstrated that a 4-week treatment with *Lactobacillus plantarum* DSM 9843 per day resulted in reduced pain and flatulence in patients with IBS [89]. Both rectal biopsies and fecal analysis indicated that the improvement in the IBS symptoms was associated with increased L. plantarum in biopsies and decreased enterococci in fecal specimens. Similar results of IBS improvement have also been reported using probiotic mix of Bifidobacterium strains, Lactobacillus strains, and Streptococcus thermophilus [90]. Studies using high-throughput assays for fecal microbiome using infants treated with Lactobacillus containing probiotic supplements showed stability in gut microbial ecology [59]. This was further corroborated by the studies where reduced microbial diversity was associated IBD [60]. Similar results were obtained in other studies that used 16S rRNA metagenomic sequencing [61] or other techniques that indicated that probiotics-mediated microbial diversity associates gut microbiome stability [62].

(B) *Probiotic in intestinal enzymatic activities and volatile fatty acid signaling*.

B1. Probiotics and intestinal enzymatic activities: probiotics facilitate beneficial effects through microbial enzymatic activities. Of particular interest is the inhibition of *β*-glucuronidase activities coming from harmful bacteria. These bacteria hydrolyze glucuronidated metabolites through *β*-glucuronidase activity in the intestinal lumen resulting in toxic metabolite formation and intestinal damage. Studies suggest probiotic supplements containing *B. longum* facilitate decrease in *β*-glucuronidase activity and associated aberrant crypt formation and colon carcinogenesis [63]. Probiotic interactions with bile acids in the luminal compartment of the intestine promote bile acid metabolism and cholesterol absorption. Bile salt hydrolase (BSH) produced by the probiotics participates in the first reaction of the deconjugation of biliary salts [64]. Due to this beneficial effects from probiotic bacteria, BSH activity has been included in FAO/WHO guidelines for the evaluation of probiotics for food use [65]. Enzymatic deconjugation of bile acids by BSH from probiotics has been one of the main mechanisms of the hypocholesterolemia effect attributed to probiotics [64, 66].

B2. Probiotics and intestinal volatile fatty acid signaling: short chain fatty acids (SCFAs) are not only the key signaling molecule in enterocytes but also an important source of their energy. Together, these maintain gut health. Once absorbed, these SCFAs also stimulate cell surface receptors in several other tissues via entering systemic circulation. For example, studies using animal models and in vitro studies showed SCFAs promote intestinal secretion of polypeptide YY and glucagon-like peptide 1 and enhance satiety through interaction with G protein-coupled receptor (GPR) 41 and GPR43, respectively [67]. SCFAs are also predicted to interact with GPR43 on adipose tissues and contribute toward decreasing fat deposits leading to decreased lipolysis and inflammation and increased adipogenesis and leptin release. On the other hand, acetate, propionate, and butyrate could also increase peroxisome proliferator-activated receptor (PPAR)-*γ*-mediated adipogenesis possibly through GPR43. Towards immunomodulation, it is hypothesized that propionate and butyrate may also reduce the secretion of proinflammatory cytokines and chemokines, thereby reducing local macrophage extravasation, and hence, reduce inflammation [67]. Another mechanism includes promotion of insulin sensitivity and decreased lipid accumulation through beta-oxidation by SCFAs through the activation of AMP-activated protein kinase by SCFAs in muscles [67]. Therefore, SCFAs play an important role in the regulation of the overall energy homeostasis and metabolism in human body. Studies suggest probiotics promote increased production of SCFAs as determined through increased fecal acetate, propionate, and butyrate in probiotics in treated group compared to nontreated group. Studies also show continued production of some of these fatty acids in the probiotic-treated groups even after the cessation of probiotic treatment when compared with nontreated group [68].

### 4.5. Modulation of Host Immunobiology by Probiotics

Probiotics and intestinal modulation of immune system: probiotics are reported to modify the intestinal immunity through altered responsiveness of the intestinal epithelia and the immune cells to gut microbes present in the intestinal lumen. Several tools and techniques ranging from specific culture-dependent methods to metagenomic sequencing are used to determine the impacts of probiotics on the composition, diversity, and function of the gut microbiome [69].

Toll-like receptors (TLRs) are a family of pattern recognition receptors dedicated for recognizing an array of microbial components called pathogen-associated molecular pattern (PAMPs). Mammal TLRs include 11 members (TLR1–TLR11) where different TLRs recognize different specific components of microbes. These microbial components bind to the extracellular leucine-rich repeat regions of different TLRs, thereby activating the TLR signaling. In humans, while TLR1, TLR2, TLR4, TLR5, TLR6, and TLR10 are expressed on the mammalian cell membrane and recognize bacterial surface-expressed PAMPs, TLR3, TLR7, TLR8, and TLR9 are present intracellularly on surfaces of endosomes of mammalian cells and recognize bacterial/viral nucleic acid-based PAMPs. Activation of majority of TLR facilitates recruitment of *Myd*-88 followed by activation of MAPK and NF-*κ*B signaling ultimately leading to inflammatory cytokine secretion. TLR3 activation, however, leads to TIR-domain-containing adapter-mediated interferon production. TLR9 signaling on the other hand is responsible for the anti-inflammatory effect of probiotics [70]. Studies suggest probiotics suppress intestinal inflammation through several TLR signaling-targeted mechanisms including through downregulation of the expression of TLRs and through the secretion of metabolites capable of inhibiting extracellular proinflammatory cytokines such as TNF-*α* or through intracellular inhibition of the transcription factor NF-*κ*B responsible for the expression of inflammatory cytokines in enterocytes [71]. Other mechanisms through which probiotics suppress inflammation involve suppression of IL-12 production by the host cells [72] and through reinforcing epithelial barrier functions promoting tight-junction formation [73]. Probiotics also facilitate TLR-mediated T-helper 1 cell differentiation, thereby also augmenting antibody production, increasing phagocytic and natural killer cell activity, and upregulating anti-inflammatory cytokine interleukin- (IL-) 10 and transforming growth factor beta while concurrently downregulating proinflammatory cytokines TNF-*α*, IFN-*γ*, and IL-8 [74, 75]. Probiotic Bifidobacterium longum spp. longum 35624 has been studied in detail and show increased levels of IL-10 in the serum of volunteers orally administered with the probiotic [76, 77]. This strain also showed reduced C-reactive protein levels in patients with ulcerative colitis (UC) [78]. Molecular basis for these effects shows engagement of dendritic cell-mediated induction of regulatory T cells which are the main source for IL-10 production. Indeed, the probiotic also showed dampening of the inflammatory response towards common pathogen such as *Salmonella typhimurium* where the exopolysaccharide coat was shown to have the anti-inflammatory effects [79]. In addition, the probiotic strain of *Faecalibacterium prausnitzii* was reported to have protective effects in patient with IBD through induction of IL-10 which was through its impact on both human and murine dendritic cells, thereby preventing the infare of chronic inflammation [80].

### 4.6. Probiotics in Ulcerative Colitis

Compared to CD, probiotics are reported to have more beneficial effects in UC [81]. For example, probiotics are noted to desensitize the abilities of dendritic cells thereby making them less reactive to gastrointestinal bacteria. Since it is suggested that one of the etiologies of UC include gut dysbiosis, these conditions with more aggressive bacteria may stimulate the mucosal immune system and can cause chronic inflammatory responses. Probiotics are speculated to work through changing the existing microbiota by replacing the aggressive bacteria with nonaggressive bacteria thereby mitigating the inflammatory responses [91]. There is also a lack of convincing data supporting the efficacy of some of the probiotics such as *Lactobacillus* in the prevention and/or treatment of UC in humans. However, *Escherichia coli* Nissle is one of the promising probiotics that is recommended by the ECCO guidelines as a maintenance therapy for UC. Moreover, the use of *Escherichia coli* Nissle may also worsen the remission of ulcerative colitis in some individuals [92]. A comparison of probiotic milk (*Bifidobacterium breve*, *Bifidobacterium bifidum*, and *Lactobacillus acidophilus)* versus placebo is reported to have significantly improved change in clinical activity index and histological score in the probiotic group during the evaluation of the induction of remission [93]. Another probiotic combination of 8 strains of bacteria marketed as VSL #3 is claimed to improve IBS, ulcerative colitis, and pouchitis conditions where studies have suggested safety and efficacy with the use of VSL #3 in the maintenance of ulcerative colitis [82, 94]. The mechanism of action for these effects includes among others reduction of TNF-*α*, interferon-*γ*, improvement of the colonic barrier function, conversion of linoleic acid into conjugated linoleic acid, inhibition of TNF-*α*-induced IL-8 secretion, and upregulation of mucin expression [83].

### 4.7. Probiotics in Crohn's Disease (CD)

Reports indicate that the intestinal microbiome plays a key role in the development of Crohn's disease. Since the use of immunosuppressors have complications resulting from host's vulnerability to infections, probiotics have been sought as safe alternative due to high safety profile as maintenance therapy alternates after drugs or surgery failed [95].

In a prospective double-blind study, patients who were operated for Crohn's disease were randomly selected and administered with 12 billion CFU of lactobacillus. No significant effectiveness was noticed in lactobacillus group towards the prevention of relapse as suggested in this study. Data on the effectiveness of probiotics in the treatment of IBD is limited. However, it is speculated that the severity of Crohn's disease may be improved with the administration of probiotics in the early stages as it can reduce the permeability of the gastrointestinal mucosa [96].

## 5. Probiotic Engineering in IBD

### 5.1. Probiotic Engineering in IBD Therapeutics

Both US and CD in IBD share symptoms including ulceration or tissue damage in the GI tract [97, 98]. As gut microbiota play crucial role in the onset of all these symptoms [99–101], microbiota-derived signaling molecules are now emerging as the facilitator of the altered intestinal barrier function and inflammation. Current therapies through anti-inflammatory drugs are often expensive and more importantly ineffective in the long run. The use of probiotics provides an alternate which rebalances the gut microflora shifting the balance from pro- to anti-inflammatory state. The most common strains currently available as probiotics are (1) the *Bifidobacterium* species, (2) *Enterococcus faecium*, (3) the *Lactobacillus strains*, (4) *Saccharomyces boulardii*, (5) the *Bacillus species*, and (6) *Pediococcus*, and all of them are found to have beneficial health effects [51, 102]. Molecular mechanisms of the beneficial effects by these probiotics include (a) production of stimulatory signaling proteins, quorum sensing signaling inhibitors, butyrate production, immunoglobulin A formation, and SCFA; (b) decreased production of proinflammatory cytokines such as tumor necrosis factor alpha and interleukin 8; (c) increased expression of mucin 2; and (d) increased upregulation of defensin(e) increased autophagy [103, 104]. Though studies on probiotics in both animal models of IBD and clinical results in IBD patients are encouraging, theoretical risks have been described in some case reports, clinical trial results, and experimental models. These include systemic infections, deleterious metabolic activities, excessive immune stimulation in susceptible individuals, gene transfer, and gastrointestinal side effects [105].

Probiotic engineering uses bacterial strains well suited for colonization in the GI track with an ability to produce a desired therapeutic molecule in situ. These therapeutic molecules are based on the study mentioned in the above-mentioned probiotic signaling pathways. Probiotic engineering allows formation of robust probiotic strains with enhanced functional properties for not only targeted control of enteric pathogenic microorganisms but also for specific intervention in IBD. Through probiotic engineering, bacteria are engineered as delivery vehicles to produce one or multitude of therapeutic biomolecules with a potential to treat intestinal inflammation and avoid common complications and side effects emanating from current therapies.

Commonly, in probiotic engineering, bacteria or yeasts are genetically engineered with the genes for therapeutic substances acting as anti-inflammatory agents [106–108]. These genetically modified probiotics either constitutively or in an inducible manner express the therapeutic proteins [109]. The use of inducible systems is not only preferred over the constitutive systems because of less adverse impact but also gaining attention. This is because it is easy to control the in situ therapeutic biomolecule production and help avoid overdosing the therapeutic biomolecule that might have undesirable effect at an elevated concentration. Recently, study used engineered *E. coli* Nissle 1917 to produce an extracellular matrix containing all three trefoil factors to treat inflammation [14]. One of the strategies used in probiotic engineering include the use of xylan-inducible system from Bacteroides species, a dominant gut microbiome. A xylan-inducible system in *B. ovatus* was able to produce human keratinocyte growth factor 2 and transforming growth factor *β*. Both these biomolecules are important for maintaining intestinal integrity through intestinal epithelial cell proliferation [110]. On the other hand, *Lactococcus lactis* has been engineered to produce IL-10 in a regulated manner under the control of inducible promoter xylose-inducible expression system (XIES) to genetically modify this bacterium, regulating the expression of the cytokine by modifying the concentration of xylose present [111].

IL-10 is an anti-inflammatory cytokine; the main function of which is to inhibit the effector T-cell functions, thereby limiting the inflammatory responses from pathogens [112]. *Lactococcus lactis* has been engineered to produce IL-10 in a regulated manner under the control of inducible promoters' xylose inducible expression system (XIES) to genetically modify this bacterium and regulate the expression of this cytokine by modifying the concentration of xylose as inducer in the system present [111]. Under physiological conditions, it also plays an important role in wound healing, autoimmunity, and homeostasis signals through two receptor complex IL-10 receptor 1 and IL-10 receptor-2 proteins. IL-10 binding to the IL-10 receptors activates JAK-STAT3 pathways [113]. Studies in both animal models and humans suggest the involvement of IL-10 in many diseases that involved mutations in the IL-10/IL-10R axis [114, 115]. These diseases occur in patients who have mutations either in the IL-10 gene itself or have through its epistatic interaction with genes within the IL-10/STAT3 signaling pathway that contribute to the risk of developing pediatric IBD. One interleukin- (IL-) 10 genetic variation, rs304496, is associated with risk for pediatric IBD [116, 117]. IL-10 dysfunction was also observed in a subgroup of pediatric IBD patients having higher IL-1*β* expression in activated immune cells and macroscopically affected intestinal tissue. Reduced IL10RA expression was detected in peripheral blood mononuclear cells, and a subgroup of pediatric IBD patients exhibited diminished IL-10 responsiveness. Therefore, IL-10 treatment has now shown promise in clinical trials for the treatment of IBD where IL-10 restricts excessive immune responses during intestinal inflammation [115]. Specifically, it is the IL-10 gene and the epistatic interactions between genetic variants within the IL-10/STAT3 signaling pathway that contribute to a higher associated risk for pediatric IBD. Using two mouse models, it was reported that the therapeutic dose of IL-10 can be reduced by localized delivery of a bacterium genetically engineered through recombinant DNA technology to secrete this cytokine. Administration of IL-10-secreting *Lactococcus lactis* caused a 50% reduction in colitis in mice treated with DSS and prevented the onset of colitis in IL-10(-/-) mice. Mechanistically, IL-10 controls IFN*γ*-secreting CD4+ T cells in humans and identifies IL-1*β* as a potential classifier for a subgroup of IBD patients. This approach may lead to a better method for cost-effective and long-term management of IBD in humans. Data presented from two different studies performed by IBD Cooperative Study Group suggest that 23.5% of patients receiving a dose of 5 *μ*g/kg of IL-10 had improvement compared with placebo [118, 119].

Networks of cytokines have been implicated in both the forms of IBD. For example, interleukin- (IL-) 12, IL-18, IL-21, and IL-27 transcript levels were significantly higher than in control than the IBD cohorts [120–122]. Out of these, IL-27, a type I cytokine, plays an important role in infectious disease, autoimmunity, and cancer in a variety of organs, including the central nervous system, lung, skin, and gastrointestinal tract [123]. IL-27 signals through a heterodimeric receptor consisting of IL-27R*α*. In animal model, treatment with IL-27 attenuates experimental colitis through the suppression of the development of IL-17-producing T helper cells. Interestingly, engineered IL-27-producing *L. lactis* proved more effective than both the IL-10 producing counterpart and systemic administration of IL-27 in colitis mouse models [124, 125]. It was shown that this engineered strain increased the production of IL-10 in the intestinal epithelium, contributing to the effectiveness against colitis. Treatment with IL-27 attenuates experimental colitis through the suppression of the development of IL-17-producing T helper cells in TNBS-induced colitis model even after active colitis was established. These results suggest new possible therapeutic approaches for IBD, including Crohn's disease and ulcerative colitis. Multiple other studies have also implicated IL-27 as a candidate gene for IBD [126, 127]. Mucosal administration of IL-27 synthesized in situ by a food-grade bacterium improved survival and significantly decreased disease activity as determined through the analysis of colon and small intestine histopathology scores and proinflammatory gene expression within the intestine in a mouse model of enterocolitis induced by T cell transfer [124]. The treatment effects in this study were both T cell- and IL-10-dependent; however, mucosal delivery of IL-27 was found to be more efficacious than direct mucosal delivery of IL-10 by the bacteria [128]. A possible explanation was that IL-27 induces higher levels of endogenous IL-10 in the intestine and mesenteric lymph nodes than could be achieved by the bacteria-producing IL-10 in situ. Interestingly, mucosal delivery of IL-27 was also more effective than systemic administration of recombinant murine IL-27 in this study. Consistent with previous literature, IL-27 treatment significantly decreased the expression of ROR*γ*t (retinoic-acid-receptor-related orphan nuclear receptor gamma) in the colons of enterocolitis mice, thereby decreasing the expression of both IL-17A and IL-17F. This study went further to show that IL-27 treatment also reduced disease activity in dextran sulfate sodium- (DSS-) induced colitis model in mice. Subcutaneous treatment with IL-27 in an acute chemically induced model of colitis using 2,4,6-trinitrobenzenesulfonic acid (TNBS) was also reported to be protective with improved colonic macroscopic and histopathology scores and reductions in several of the same proinflammatory cytokines previously reported [44] including IL-6, TNF-*α*, IL-17A, and IL-1*β* [129].

**Figure 1:**
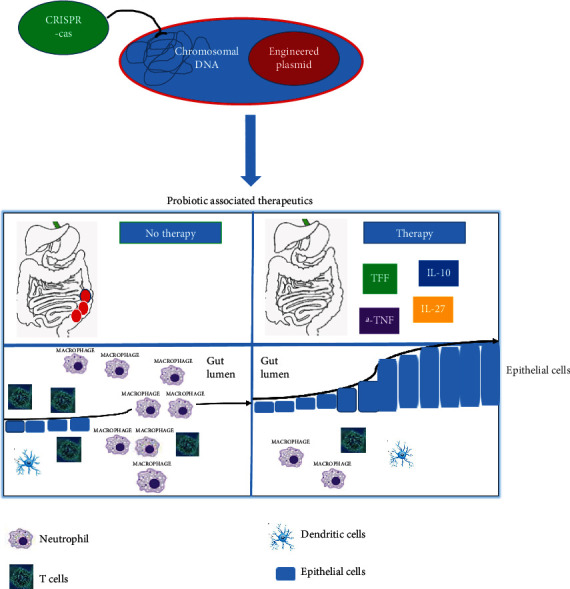
A model of cytokine-driven/targeted probiotic engineering. The promotor-driven expression of anti-inflammatory cytokines or antimicrobial peptides by the engineered probiotics may facilitate epithelial wound repair and the differentiation/reformation of mucosal barrier through inhibiting the infiltration of inflammation causing immune cells, thereby decreasing both intestinal and systemic inflammation.

Probiotic engineering therefore holds great promise in having long-term therapeutic potential for IBD (Figure 1). For example, *Lactococcus lactis* has been the focus of probiotic engineering for quite some time. Genetic engineering led these modified bacteria to express for example IL-10, the anti-inflammatory cytokine (166). These engineered bacteria also increase therapeutic bioavailability through direct contact with the mucosa (142). However, a phase II clinical trial indicated that engineered IL-10-expressing *L. lactis* was sometimes ineffective in inducing mucosal wound healing. Interleukin-27 was also engineered into *L. lactis* where the gene was inserted into *L. lactis* to facilitate a localized delivery strategy. Such strategy showed that IL-27 can successfully reduce ulcerative colitis in mice. Furthermore, this localized delivery using *L. lactis* to the intestines was more effective than a systemic administration of IL-27 (164).

Interleukin 35 (IL-35) is an anti-inflammatory cytokine from the IL-12 family and produced by regulatory lymphocytes and plays an important role in immune suppression (167, 168). IL-35 plays a pivotal role in the development and the function of both regulatory B (Bregs) and T cells (Tregs). IL-35 functions as a new anti-inflammatory factor for IBD and other immune diseases (169). Levels of IL-35 and IL-35-inducible Treg (iTR35) cells are dysregulated in these inflammatory conditions. Therapeutic potential of recombinant IL-35 protein was assessed in DSS-induced colitis mouse model. Recombinant IL-35 protein could slow down the pathologic process in mouse model by decreasing the infiltrations of macrophages and CD4+ T and CD8+ T cells and by promoting the infiltration of Treg cells. Further analysis demonstrated that IL-35 recombinant protein regulated inflammation through promoting the secretion of IL-10 and inhibiting the expression of proinflammatory cytokines such as IL-6, TNF-*α*, and IL-17 (169).

Several studies also support the involvement of trefoil peptides in mucosal surface protection and repair after injury. Trefoil factors (TFFs) include a family of three mucin-associated peptides (TFF1, TFF2, and TFF3) that are widely expressed in a tissue-specific manner in the gastrointestinal tract (170–172). Trefoil factors (TFF) and antitumor necrosis factor-*α* (TNF-*α*) nanobodies (single-domain antibody fragments) are other therapeutic substances that have been constitutively expressed in *L. lactis* and tested in DSS-induced colitis in mice (173). The former are peptides that are differentially produced in specific sections in the gastrointestinal tract and have protective and reparative properties on the intestinal epithelium (174). Specifically, TFF-1 and TFF-2 are produced in the stomach and duodenum in mucus-producing cells, while TFF-3 is produced in the small and large intestines, predominantly by goblet cells. The peptides produced in situ by *L. lactis* were considerably more effective at healing colitis than the oral or rectal administration of the purified TFF. *E. coli* Nissle 1917 (EcN) used to genetically engineer TFF was able to secrete the curli-fused TFFs in vitro and in vivo and was nonpathogenic. Genetically engineered EcN produced an extracellular matrix containing all three trefoil factors to treat inflammation. Protective effects of the engineered EcN against DSS-induced colitis in mice were associated with barrier function reinforcement and immunomodulation (145). Another example of probiotic engineering involved trefoil factors (TFF), the gastric peptides involved in protecting and stabilizing the gastrointestinal mucosa (175). The TFF-producing *L. lactis* showed better results in reducing colitis than administering 1200-fold higher dose of TFF directly (142, 175).

Tumor necrosis factor (TNF) is a multifunctional proinflammatory cytokine secreted by inflammatory cells involved in inflammation-associated pathophysiological conditions of IBD (176). TNF-*α* is a ligand that binds to the TNF receptor (TNFR1) and initiates the proinflammatory and proapoptotic signaling cascades through the activation of either NF-*κ*B or MAPK pathway. Some protein-based inhibitors target the TNF-*α* molecule or its receptor, which prevents the resultant signaling pathways (177). TNF blockers are used to suppress the immune system activation by blocking the activity of TNF (178). Antibodies for this cytokine are currently used as a treatment for IBD (45, 179). Nevertheless, these treatments are expensive and are associated with systemic administration-related diverse side effects. On the other hand, *Lactococcus lactis* was engineered to secrete monovalent and bivalent murine (m)TNF-neutralizing nanobodies as therapeutic proteins, and they are proved to have the beneficial effects. These therapeutic proteins are derived from fragments of heavy-chain camelid antibodies and are more stable than conventional antibodies. Daily oral administration of nanobody-secreting *L. lactis* resulted in local delivery of anti-mTNF nanobodies in the colon and significantly reduced inflammation in mouse model of DSS-induced colitis (142). In addition, this approach was also successful in improving established enterocolitis in IL10–/– mouse (180). Excessive TNF-ɑ production has also been linked with induction of inflammation, apoptosis, and fever-associated Crohn's disease (181). Therefore, several antitumor necrosis factor- (TNF-) alpha antibodies such as infliximab, adalimumab, pembrolizumab, and nivolumab have been suggested to treat various chronic inflammatory conditions including IBD. However, direct treatment with anti-TNF-ɑ antibodies have many disadvantages not to mention severe side effects such as infusion reaction and anaphylaxis. *L. lactis* engineered to secrete anti-TNF nanobodies have been shown to prevent the systemic side effects through localized delivery. Though early in preclinical phase, these nanobodies have shown success in reducing inflammation in a mouse model of DSS-induced colitis (142).

### 5.2. Probiotic Engineering in Vaccinations

Traditional oral vaccinations have many disadvantages such as failing to induce effective immunity because of their inability to withstand the harsh environment of the stomach. Sometimes, they are also unable to specifically target the essential immune structures that induce immunity. In addition, there are also chances of reversion to a virulent state when using attenuated microbes [130]. Alternatively, probiotics can be engineered to deliver medication and vaccinations where these oral probiotic vaccinations are effective in producing immunity in the gastrointestinal tract. Engineered probiotics are also more convenient to store and are much cheaper than the traditional biologics [131]. Engineered probiotics enhance the potential of survival under undesirable environmental conditions and can be manipulated to induce tolerogenic immune response. In addition, these probiotics can be targeted to certain immune structures such as Peyer's patches [132].

### 5.3. Current Challenges in Probiotic Engineering

Although bioengineered probiotics show promise and are increasingly studied due to their convenience and effectiveness, there are several safety concerns. First and foremost, bioengineered probiotics are considered genetically modified organisms (GMO) [133], and being microbes, they pose special challenge in the approval process. Additionally, consumers are often sceptic about the safety of GMO and their impact on the environment. However, it is surely possible to create guidelines and containment strategies to prevent bacterial gene transfer. Alternatively, these bacteria could be engineered to prevent survival in the natural environment which may alleviate some of the aforementioned concerns [134]

## 6. Final Considerations and Future Direction

Probiotic engineering is the frontier of synthetic biology tailored with natural science with great promise to treat previously untreatable chronic inflammatory conditions such as IBD. Emerging technology such as CRISPR-Cas system and other genetic engineering tools has unlimited potential that can also be utilized to manipulate probiotic bacterial system to produce useful therapeutic biologics with a goal to either ameliorate and/or treat previously untreatable chronic inflammatory conditions. As new findings increase our understanding of viable bacterial strains and synthetic biology tools, in forceable future, it is highly possible to identify and characterize additional probiotic bacterial strains as potential candidates for probiotic engineering. As gut microbiome is diverse and varies in different ethnic, geographic, and cultural groups, it will be important to develop vision for personalized probiotic treatment and develop the technology thereof to make personalized probiotic options a reality. Researchers must also recognize certain performance metrices to ensure that these genetically engineered bacteria are noninvasive and have a site-specific localized action. With these in mind, the days are not far, where probiotics can be used as a safe with long-term efficacy alternate to treat IBD through the restoration of the normal gut microbiota.

## Data Availability

The data that support the findings of this study are available from the authors upon reasonable request.
